# Low vaccination and infection rate of Omicron in patients with inflammatory bowel disease: a comparative study of three unique cohorts

**DOI:** 10.3389/fpubh.2023.1115127

**Published:** 2023-06-15

**Authors:** Jing Feng, Tian Yang, Ruchen Yao, Bo Feng, Renshan Hao, Yuqi Qiao, Jinlu Tong, Jun Shen

**Affiliations:** ^1^Division of Gastroenterology and Hepatology, Key Laboratory of Gastroenterology and Hepatology, Ministry of Health, Shanghai Institute of Digestive Disease, Inflammatory Bowel Disease Research Center, Ren Ji Hospital, Shanghai Jiao Tong University School of Medicine, Shanghai, China; ^2^Department of Respirology, Baoshan Branch, Ren Ji Hospital, Shanghai Jiao Tong University School of Medicine, Shanghai, China; ^3^Department of Internal Medicine, Meipu Temporary Hospital, Shanghai, China; ^4^Department of Gastroenterology, Baoshan Branch, Ren Ji Hospital, Shanghai Jiao Tong University School of Medicine, Shanghai, China

**Keywords:** Omicron, severe acute respiratory syndrome coronavirus 2 (SARS-CoV-2), coronavirus disease-19 (COVID-19), inflammatory bowel disease (IBD), asymptomatic

## Abstract

**Background:**

The SARS-CoV-2 Omicron variant caused a large-scale outbreak of COVID-19 in Shanghai, China. Patients with inflammatory bowel disease (IBD) are at high risk of infection due to immunosuppressive interventions. We aimed to investigate the vaccination information of patients with IBD and update a vaccination guide based on a comparison of vaccination in asymptomatic carriers and healthy individuals.

**Methods:**

This retrospective study was conducted during an Omicron variant wave. We assessed the vaccination status in patients with IBD, asymptomatic carriers and healthy individuals. Factors with unvaccinated status and adverse events following vaccination were also determined in patients with IBD.

**Results:**

The vaccination rate was 51.2% in patients with IBD, 73.2% in asymptomatic carriers, and 96.1% in healthy individuals. Female sex (*p =* 0.012), Crohn’s disease (*p =* 0.026), and disease behavior of B3 (*p =* 0.029) were factors that indicated a lower vaccination rate. A significantly higher proportion of healthy individuals had received one booster dose (76.8%) than asymptomatic carriers (43.4%) and patients with IBD (26.2%). Patients with IBD received vaccination without an increased risk of adverse events (*p =* 0.768).

**Conclusion:**

The vaccination rate of patients with IBD remains much lower than that of asymptomatic carriers and healthy individuals. The COVID-19 vaccine has been found to be safe among all three groups and patients with IBD are not more susceptible to adverse events.

## 1. Introduction

The Omicron lineage of SARS-CoV-2 became globally dominant within a few days of its first detection in Africa in November 2021 because of its powerful infectivity and poor detectability by the immune system (1). It split into multiple sub-lineages, among which the BA.2 variant is spreading rapidly throughout the world and has sparked a new wave of fulminant infection in Shanghai, China. As of June 1, 2022, a total of 626,737 individuals tested positive for the Omicron BA.2 and BA.2.2 variants during the Shanghai 2022 SARS-CoV-2 Omicron variant wave, most of which were asymptomatic carriers (2, 3).

Compared with the original variant of SARS-CoV-2, Omicron variants seem to be less severe, particularly in vaccinated individuals, but still lead to a number of severe situations in individuals with low immunity. Therefore, it is necessary to implement measures to prevent highly transmissible infections. Vaccination is currently the best available tool to protect people from SARS-CoV-2 infection and appears to be particularly necessary in those with pre-existing health conditions, although SARS-CoV-2 Omicron variants are believed to be partially resistant to infection- and vaccine-induced immunity.

Inflammatory bowel disease (IBD), mainly comprising ulcerative colitis (UC) and Crohn’s disease (CD), is an immune-mediated inflammatory gastrointestinal disease with steadily increasing prevalence worldwide. Patients with IBD are frequently treated with immunomodulators, which can increase the risk of infection. Facing the omicron variant pandemic, particular attention should be paid to the prevention of infection. Major IBD societies recommend the vaccination of patients with IBD at the earliest possible stage for better protection (4, 5). In practice, not all IBD clinicians suggest vaccination during the active disease stage or immunosuppressive situation, and a number of patients with IBD still hesitate to be vaccinated for various reasons, leading to suboptimal vaccination coverage (6, 7). To date, there existed a limited number of documented cases of SARS-CoV-2 infection in patients with IBD on a global scale, with only one case of infection reported locally during this local Omicron wave. Nonetheless, the risk of Omicron variants in patients with IBD should not be underestimated and SARS-CoV-2 may be mutated to other variants in the future. Breakthrough infections in vaccinated people are noticed in asymptomatic patients with COVID-19, and vaccination strategies for patients with IBD should be dynamically adjusted according to real-world settings.

In this study, we assessed the COVID-19 vaccination rate, factors related to unvaccination, and the post-vaccination adverse events (AEs) in patients with IBD. In particular, we included a detailed comparative analysis of the vaccination status of asymptomatic carriers and uninfected healthy people during the 2022 Omicron wave. The analysis of vaccination information for three specific cohorts conveys more practical vaccination information for patients with IBD, beyond the highly transmissible Omicron BA.2 and BA.2.2 variants.

## 2. Methods

### 2.1. Study design and participants

This was a retrospective, cross-sectional study. The study was conducted during the new wave of SARS-CoV-2 Omicron variant infections in Shanghai, making it possible for us to evaluate the impact of vaccination and restrictions on public gatherings. Patients with IBD who were regularly followed up at Ren Ji Hospital, Shanghai, China between January 1, 2020, and June 1, 2022, were enrolled in this study. Patients were followed up closely during the Shanghai COVID-19 outbreak using the telephone and WeChat app. Asymptomatic carriers with Omicron variants were double-confirmed by PCR testing, and vaccination data were included via the hospital information system of Meipu Temporary Hospital, which is a designated COVID-19 treatment campus affiliated to Baoshan Branch, Ren Ji Hospital, Shanghai Jiao Tong University School of Medicine. These asymptomatic carriers were non-IBD patients and had no history of immune-related disorders. Healthy individuals were enrolled from the physical examination center of Ren Ji Hospital.

**Figure 1:**
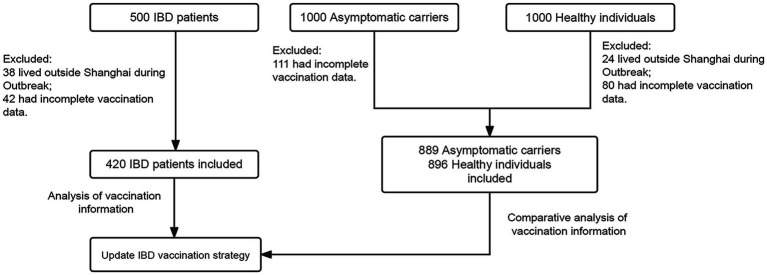
Flowchart of patients’ recruitment.

The inclusion criteria were: (i) patients with IBD diagnosed based on the European Crohn’s and Colitis Organization guidelines (8), (ii) actively followed-up *via* the hospital information system, and (iii) no history of SARS-CoV-2 variant infection. Asymptomatic carriers were double-checked by PCR testing and diagnosed according to the Corona Virus Disease-19 Prevention and Control Consensus Diagnosis and Treatment of Corona Virus Disease-19 (9^th^ edition, China) (9), which is quite similar to the Centers for Disease Control and Prevention of the US. Healthy individuals without a history of SARS-CoV-2 variant infection were confirmed by PCR testing and none of them had any other immune-related disorders. Participants with missing vaccination data or those living outside Shanghai during the regional outbreak were excluded from the study (Figure 1). The need for informed consent was waived in view of the retrospective observational nature of the study. The study protocol was approved by the Institutional Review Board of Ren Ji Hospital, Shanghai Jiao Tong University School of Medicine (KY2022-162-B). This study was conducted in accordance with the Declaration of Helsinki to protect the patients’ confidentiality of information.

### 2.2. Data collection

We conducted a survey and analysis of vaccination data for three cohorts: patients with IBD, asymptomatic carriers, and healthy individuals. Participants vaccinated with at least one dose were recognized as vaccinated, and the unvaccinated rate was documented until June 1, 2022.

For patients with IBD, demographic characteristics and data related to IBD diagnosis during their recent hospitalization were retrospectively extracted from medical records. They were actively followed up every 3 months via a hospital information system phone call and/or WeChat app. Extra follow-up was performed between May 20 and June 1, 2022, by telephone regarding the overall disease situation, the SARS-CoV-2 Omicron variant infection condition, and vaccination information, including vaccination status, number of doses, AEs within 7 days of vaccination, or factors related to unvaccination. The characteristics and vaccination information of asymptomatic carriers were summarized from medical records with concealed identification information. For healthy individuals, relevant information was obtained from medical records at the time of medical examination at our hospital.

### 2.3. Statistical analysis

Statistical analysis was performed using R version 4.0.3 and STATA version 15.0 for Windows (Stata Corp LLC, College Station, TX, United States). Normally distributed continuous variables are presented as means and standard deviations, and non-normally distributed variables are presented as medians and interquartile ranges. Categorical variables are presented as proportions. Comparisons between the two groups were made using Student’s *t*-test, Mann–Whitney U test, chi-squared test, or Fisher’s exact test, as appropriate. The proportion of unvaccinated participants was calculated in the full analysis set and compared between cohorts. An additional safety analysis was conducted in patients with IBD and healthy individuals who received at least one dose of COVID-19 vaccination. Univariate analysis was used to identify variables that may be associated with the unvaccinated status.

Sample size estimation was performed using an online sample size calculator.^1^ Based on previously reported vaccination data for patients with IBD and asymptomatic carriers (50% and 70%, respectively), we estimated the sample size with a significance level of *α* = 0.05, and the matching ratio of the two groups was set to 1:2. A ratio 1:1 was used to evaluate the sample size of asymptomatic carriers and healthy individuals. All statistical tests were two-sided, and statistical significance was set at *p* < 0.05.

## 3. Results

### 3.1. Study population

To evaluate the impact of vaccination and restrictions on public gatherings during the local outbreak of SARS-CoV-2 Omicron variant infection, only participants who lived in Shanghai during the local outbreak were included. Forty-two patients with IBD, 111 asymptomatic carriers, and 80 healthy individuals were excluded from our study owing to loss to follow-up or incomplete information records. Finally, 420 patients with IBD, 889 asymptomatic carriers, and 896 healthy individuals were included in the current analyses. Participants who received at least one dose of the vaccine were administered inactivated vaccines. The recruitment process is shown in Figure 1.

### 3.2. COVID-19 vaccination and factors related to unvaccination in patients with IBD

The characteristics of the patients with IBD included in this study are summarized in Table 1, and comparative analyses are presented between the unvaccinated and vaccinated cohorts. There were predominantly male patients (66.2%) with CD (69.8%), undergoing biological treatment during or within the last 12 months of vaccination (85.2%) with a mean age of 34.4 ± 11.1 years. None of the patients were infected with the SARS-CoV-2 Omicron variant during the Shanghai 2022 outbreak.

**Table 1:** Characteristics and factors associated with vaccination status in patients with IBD.

	All (*n* = 420)	Unvaccinated (*n* = 205)	Vaccinated (*n* = 215)	*p*
Sex				0.012
Female	142 (33.8)	82 (40.0)	60 (27.9)	
Male	278 (66.2)	123 (60.0)	155 (72.1)	
Age	34.4 (11.1)	35.2 (11.0)	33.5 (11.2)	0.123
IBD type				0.026
CD	293 (69.8)	154 (75.1)	139 (64.7)	
UC	127 (30.2)	51 (24.9)	76 (35.3)	
Extension CD^a^ (L1; L2; L3)		48 (31.2); 19 (12.3); 87 (56.5)	48 (34.5); 22 (15.8); 69 (49.6)	0.465
Behavior CD^b^				
(B1; B2; B3)		83 (53.9); 40 (26.0); 31 (20.1)	90 (64.7); 36 (25.9); 13 (9.35)	0.029
*P* ^b^		89 (57.8)	69 (49.6)	0.200
Surgical history	180 (42.9)	90 (43.9)	90 (41.9)	0.746
Biological therapy				0.357
IFX	231 (55.0)	117 (57.1)	114 (53.0)	
ADA	8 (1.90)	4 (1.95)	4 (1.86)	
VDZ	86 (20.5)	39 (19.0)	47 (21.9)	
UST	33 (7.86)	20 (9.76)	13 (6.05)	
Not in use^c^	62 (14.8)	25 (12.2)	37 (17.2)	

Values represent the mean (standard deviation) or *n* (%). ^a,b^Subtypes of montreal classifications; ^c^no use of biologics in the 12 months prior to vaccination. CD, Crohn’s disease; UC, ulcerative colitis; P, perianal lesion; IFX, infliximab; ADA, adalimumab; VDZ, vedolizumab; UST, ustekinumab.

Among 420 patients, 215 (51.2%) received at least one dose of the vaccine. Compared with vaccinated patients, those who remained unvaccinated were more likely to be female (odds ratio [OR] 0.58, 95% confidence interval [CI] 0.39–0.87, *p* = 0.012). As a subtype of inflammatory bowel disease, patients with CD had a lower vaccination rate than patients with UC (47.4% vs. 59.8%, *p =* 0.026). In addition, patients with CD with the disease behavior of B3, a penetrating type of Montreal classification which indicates a more severe condition, appeared to be more reluctant to be vaccinated (*p =* 0.029) (Table 2). In our study, most of the patients (*n* = 358, 85.2%) underwent biological therapy. Although the concern regarding immunosuppression of biologics seems to affect the vaccination compliance of patients, patients receiving biological agents showed no significant difference in vaccination rates (*p =* 0.357).

**Table 2:** Characteristics and factors associated with vaccination status in Crohn’s disease and ulcerative colitis.

	CD			UC		
Characteristics	Unvaccinated (*n* = 154)	Vaccinated (*n* = 139)	*p*	Unvaccinated (*n* = 51)	Vaccinated (*n* = 76)	*p*
Age	32.9(8.62)	30.6 (9.40)	0.026	42.3 (14.1)	38.9 (12.3)	0.172
Sex			0.003			0.707
Female	60 (39.0)	31 (22.3)		22 (43.1)	29 (38.2)	
Male	94 (61.0)	108 (77.7)		29 (56.9)	47 (61.8)	
Surgical history	82 (53.2)	76 (54.7)	0.898	8 (15.7)	14 (18.4)	0.873
Times vaccinated (1; 2; 3)	11 (7.91); 65 (46.8); 63 (45.3)			4 (5.26); 25 (32.9); 47 (61.8)		
Extension CD^a^			0.465			
L1	48 (31.2)	48 (34.5)				
L2	19 (12.3)	22 (15.8)				
L3	87 (56.5)	69 (49.6)				
Behavior CD^b^			0.029			
B1	83 (53.9)	90 (64.7)				
B2	40 (26.0)	36 (25.9)				
B3	31 (20.1)	13 (9.35)				
P	89 (57.8)	69 (49.6)	0.200			
Biologic therapy			0.894			0.313
IFX	102 (66.2)	98 (70.5)		15 (48.39)	16 (51.61)	
ADA	4 (2.60)	4 (2.88)		0	0	
VDZ	14 (9.09)	12 (8.63)		25 (41.67)	35 (58.33)	
UST	20 (13.0)	13 (9.35)		0	0	
Not in use^c^	14 (9.09)	12 (8.63)		11 (30.56)	25 (69.44)	

Values represent the mean (standard deviation) or *n* (%). ^a,b^Subtypes of Montreal classifications; ^c^no use of biologics in the 12 months prior to vaccination. CD, Crohn’s disease; UC, ulcerative colitis; P, perianal lesion; IFX, infliximab; ADA, adalimumab; VDZ, vedolizumab; UST, ustekinumab.

**Figure 2:**
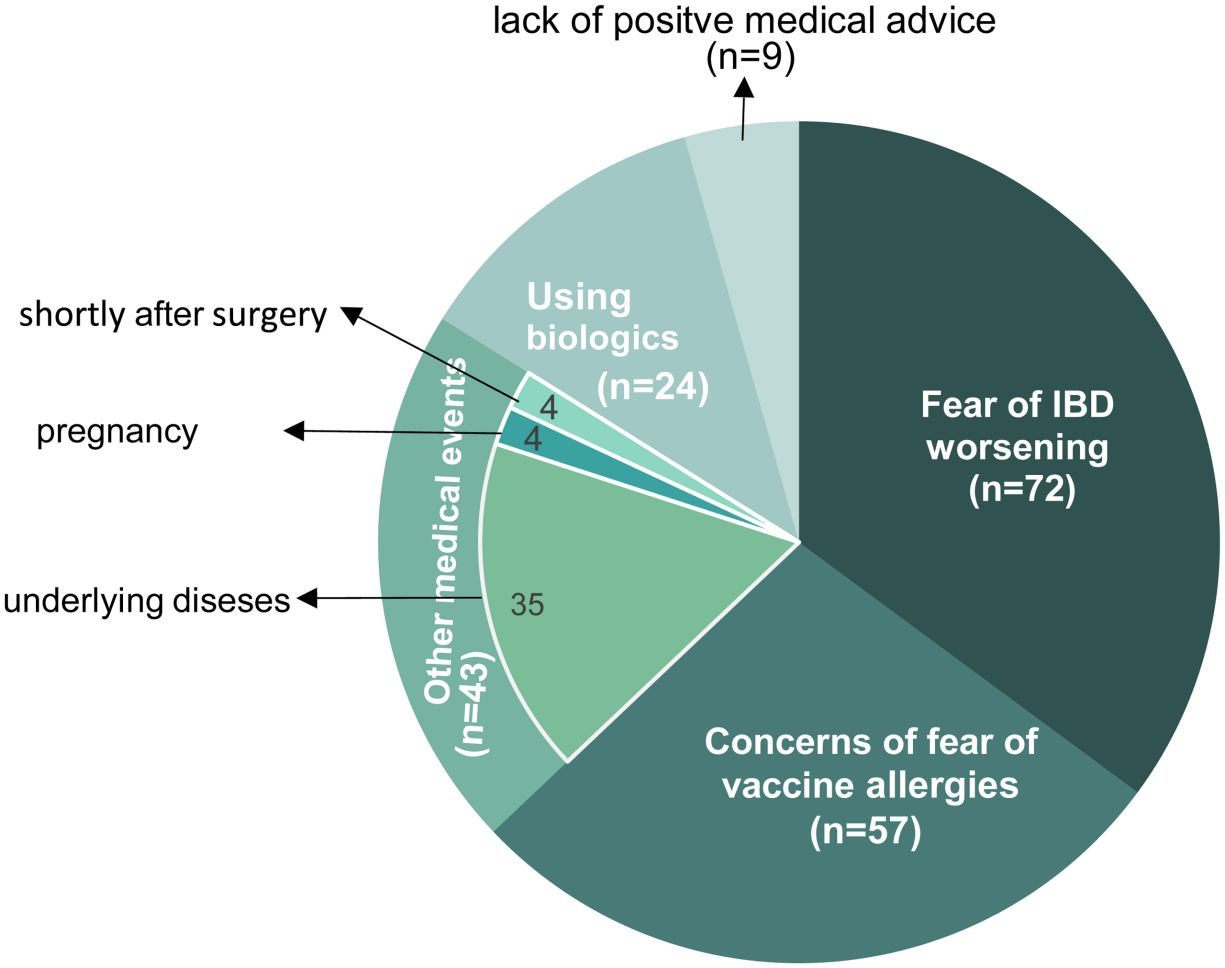
Reasons for remaining unvaccinated in patients with IBD.

Among the 205 patients who remained unvaccinated, the majority (n = 72, 35.1%) refused or delayed vaccination due to fear of IBD aggravation, 57 (27.8%) were concerned about vaccine allergies, 43 were temporarily unable to get vaccinated due to other events such as pregnancy, 24 (11.7%) were worried about the interaction between vaccine and biologics, and 9 (4.4%) claimed lack of positive medical advice (Figure 2).

### 3.3. Comparison of COVID-19 vaccination among patients with IBD, asymptomatic carriers and healthy individuals

**Figure 3:**
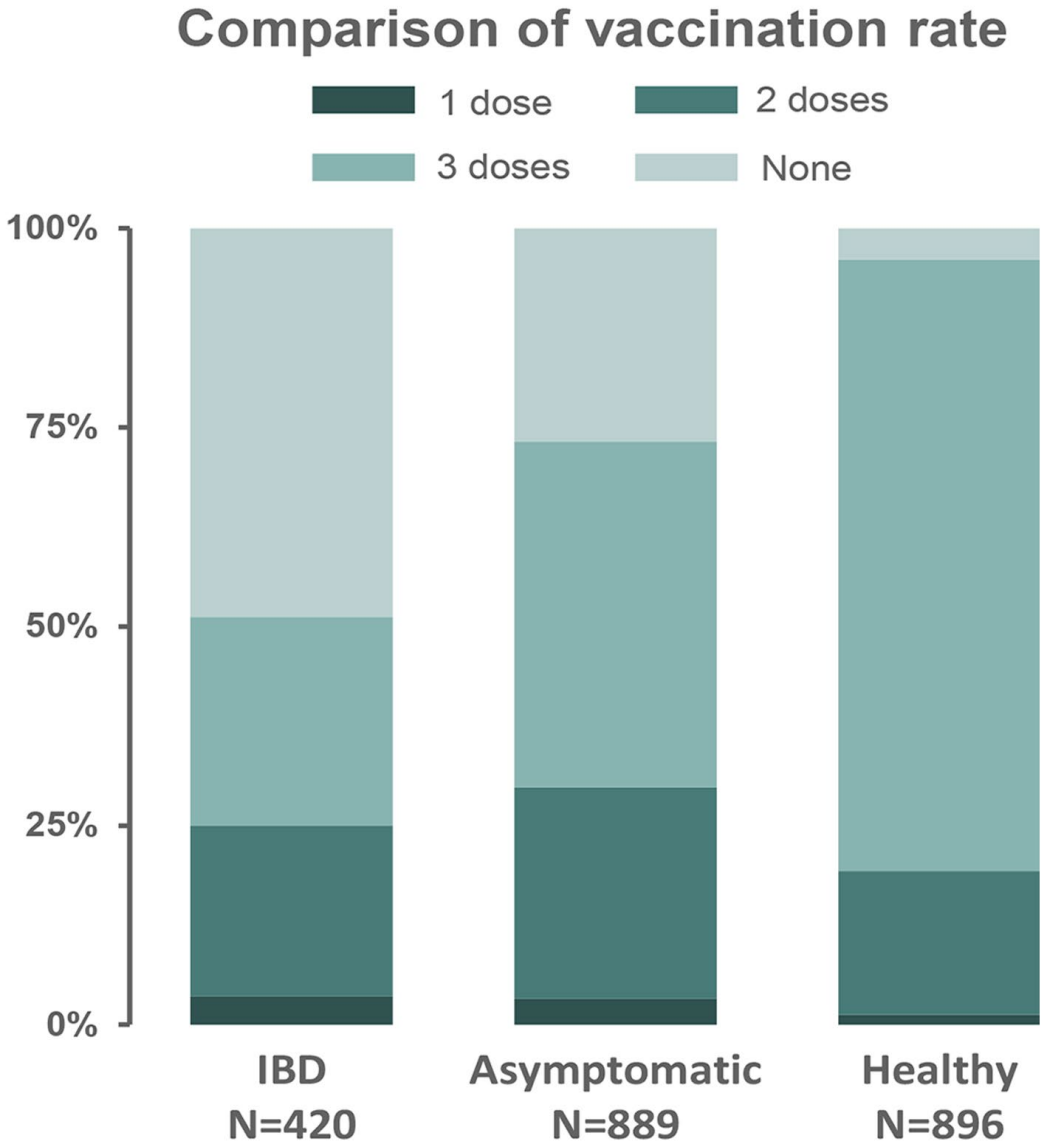
Comparison of vaccination rate among three populations.

To comprehensively evaluate the protective impact of vaccination on COVID-19 disease control across multiple populations, vaccination data were also collected and analyzed from asymptomatic carriers and healthy individuals during a local outbreak of SARS-CoV-2 Omicron variant infection (Supplementary Table S1). Of 889 asymptomatic carriers, 651 (73.2%), and 861/896 healthy individuals (96.1%), were vaccinated with at least one dose within 12 months before the outbreak, which indicated that the vaccination rate of asymptomatic carriers was significantly lower than that of healthy individuals (*p <* 0.001). There was no significant difference in age, but a significant difference in sex (OR 1.34, 95% CI 1.11–1.62, *p =* 0.003) between the two groups, indicating that men were more likely to be infected with Omicron. Moreover, patients with IBD had the lowest vaccination rate among the three groups (Figure 3). In the subgroup analysis, healthy individuals had a higher proportion of one booster dose (76.8%) than asymptomatic carriers (43.4%) and patients with IBD (26.2%) (*p <* 0.001), while the fully vaccinated individuals were similar in proportion among healthy individuals (18.1%), asymptomatic carriers (26.5%), and patients with IBD (21.4%).

### 3.4. Safety analysis of COVID-19 vaccine In patients with IBD and healthy individuals

A total of 215 patients with IBD and 861 healthy individuals who had received at least one dose of the COVID-19 vaccine were included in the safety analysis after elimination of missing data or reluctance to disclose health information. Thirty-one patients with IBD and 115 healthy individuals reported at least one adverse event after vaccination, accounting for 14.4% and 13.4%, respectively. No statistically significant difference was detected in the incidence of AEs (*p =* 0.768) (Supplementary Table S2).

The most common AEs of the two groups were “Fatigue” and “Injection-site pain” (Table 3). Most injection-site or systematic AEs were transient and could be resolved without medication. However, six patients with IBD suffered from aggravation of diarrhea, and one developed gastrointestinal bleeding 1 week after vaccination, all of whom recovered smoothly without hospitalization.

**Table 3:** Adverse events in patients with IBD and healthy individuals.

Adverse events	IBD (*n* = 31)	Healthy (*n* = 115)
Local		
Injection-site pain	5	61
Systemic		
Fatigue	11	26
Fever	1	14
Headache	5	9
Somnolence	4	24
Muscle aches	1	2
Nausea	1	1
Cough	2	2
Allergic reaction	3	3
Other*	**10**	**6**

*Six patients with IBD experienced exacerbation of diseases, such as diarrhea. Four patients with IBD presented with elevated blood pressure, gastrointestinal bleeding, swollen feet, and a 2-cm increase in breast nodules. Among the healthy individuals, one had elevated blood pressure, blood glucose, and lipids, two had irregular menstruation, one had diarrhea, and one had swollen thighs.

## 4. Discussion

The SARS-CoV-2 Omicron variant is highly transmissible and may escape vaccine-induced immunity, thereby leading to the rapid spread of COVID-19 worldwide. Thus, vaccination and isolation are necessary, especially for immunosuppressed patients. Our study was performed during the outbreak of SARS-CoV-2 Omicron variant in Shanghai, China to investigate the vaccination status of patients with IBD and compare the vaccination data between asymptomatic carriers and healthy individuals. To the best of our knowledge, no previous study has reported the differences in vaccination between patients with IBD and other non-IBD populations, including infected and non-infected individuals.

Although surprisingly few patients actually got SARS-CoV-2 Omicron variant infection during the local outbreak, the vaccination coverage of Chinese patients with IBD is of concern. Only 51.2% of patients with IBD enrolled in our study had been vaccinated, whereas vaccination rates in other countries were above 60% and even up to 95% (7, 10, 11). Our investigation indicated that women and patients with CD and relatively severe disease status were more reluctant to be vaccinated—possibly because they worried that this might aggravate their condition, which mirrors the findings of similar studies in other countries (5, 10, 12). As revealed in our study, “Fear of IBD aggravation” is the primary concern of patients with IBD. Indeed, there appears to be a widespread concern among patients with autoimmune diseases that their illnesses may be exacerbated as a result of post-vaccination AEs (13–15). Suspicion of vaccines in these patients usually stems from the misconception that the vaccines have not been studied sufficiently, despite their safety and effect have been demonstrated widely (16–18). To address these concerns, healthcare providers and IBD specialists should cooperate to proactively inform patients about the benefits of vaccination in their health, such as creating health knowledge brochures and holding vaccination lectures to dispel any misconceptions or myths that patients may have about vaccines and their potential effects on IBD.

Although the vaccination rate of patients with IBD was significantly lower than that of asymptomatic carriers and healthy individuals, no SARS-CoV-2 Omicron variant infection of IBD was reported in this local outbreak. It has also been demonstrated that patients with IBD have no increased risk of SARS-CoV-2 infection compared with the general population (19). This is probably the result of these patients’ education to avoid gatherings, alongside their tendency to self-protect more than the general population and avoid contact with high-risk populations. Nevertheless, the vaccine does prevent infections in patients with IBD. Since asymptomatic carriers have significantly lower vaccination rates than healthy individuals, the vaccine could reduce the risk of SARS-CoV-2 Omicron variant infection, despite the pronounced ability of the Omicron variant to escape being detected from the immune system. Several large cohort studies have pointed out and provided evidence that a booster vaccine could more effectively prevent the infection of Omicron variant (20). Besides, we observed an infection sex bias, with men being more susceptible than women, which confirmed the findings of other studies (21, 22). Hence, improved vaccine coverage, especially among males, is warranted.

However, quite a few patients with IBD still show a high level of vaccine reluctance and safety concerns. Previous studies on other vaccine types have considered inactivated vaccines to be safe for patients with IBD regardless of their immunosuppressive therapies (23, 24), which was further confirmed in this real-world COVID-19 vaccine study. We found that the incidence of COVID-19 vaccine AEs was similar in patients with IBD and healthy individuals, both of whom did not exceed 15%. The predominant reported AEs of healthy individuals and patients with IBD were mostly common, such as fatigue, injection-site pain, and headache, which can be relieved spontaneously within a week. Patients with IBD were not at an increased risk of developing AEs or more serious AEs after vaccination. To date, the Shanghai 2022 Omicron BA.2 infection has not yet ended, while Omicron BA.5 has already raised a new wave of infections worldwide. To better combat the new wave of the epidemic, on the one hand, patients with IBD should further strengthen their personal protection and receive vaccination whenever possible, especially men who are at a higher risk of infection. On the other hand, the government and healthcare providers should educate patients with IBD more about the efficacy of vaccines and offering a special AEs monitoring service may be a good way to allay their concerns about vaccines. Continuously developing new COVID-19 vaccines and improving the existing vaccines is also essential.

In conclusion, the COVID-19 vaccine is generally safe and effective in providing resistance against SARS-CoV-2 Omicron variant infection; however, the vaccination rate was rather low in patients with IBD. Females and patients with severe CD were less likely to accept vaccination, while males were at a higher risk of infection. It is essential for healthcare and IBD specialists to proactively convince patients with IBD of the importance and safety of vaccines and encourage them to intensify their personal protection to minimize their infection risk in face of the successive waves of COVID-19.

This study has several strengths, including a large sample size and multidimensional analysis. To our knowledge, this is currently the first largest real-world study to perform a comparative analysis of vaccination data from three unique cohorts in the same Omicron variant exposure environment: patients with IBD, asymptomatic carriers, and healthy individuals. The study provides important insights into the vaccination coverage and attitudes towards vaccination among patients with IBD in China. However, there were also some limitations to our study. First, all the participants were from a single center in Shanghai, which may lead to a regional bias. Second, there has been a lack of vaccination data for patients with IBD infected with Omicron variant. Third, the data on AEs are insufficient owing to mission information or reluctance to disclose health information. The information collected in our study was not comprehensive enough because of the tight timelines of the outbreak and the limited resources allocated to the creation of the data collection and reporting system.

## Data availability statement

The raw data supporting the conclusions of this article will be made available by the authors, without undue reservation.

## Author contributions

JF contributed significantly to perform the research and manuscript preparation. TY and RY participated in data collection. JF and BF analyzed and interpreted the clinical data. RH helped analyze part of the data. YQ helped create tables and figures. JT helped revise the manuscript. JS conceived the study and critically reviewed the content of the paper and supervised the project. All authors contributed to the article and approved the submitted version.

## Funding

This work was supported by grants from Shanghai Science and Technology Innovation Initiative (21SQBS02302) and Cultivated Funding for Clinical Research Innovation, Ren Ji Hospital, Shanghai Jiao Tong University School of Medicine (RJPY-LX-004).

## Conflict of interest

The authors declare that the research was conducted in the absence of any commercial or financial relationships that could be construed as a potential conflict of interest.

## Publisher’s note

All claims expressed in this article are solely those of the authors and do not necessarily represent those of their affiliated organizations, or those of the publisher, the editors and the reviewers. Any product that may be evaluated in this article, or claim that may be made by its manufacturer, is not guaranteed or endorsed by the publisher.

## Footnotes

^1^ http://www.powerandsamplesize.com/
